# Glass Imprint Templates by Spark Assisted Chemical Engraving for Microfabrication by Hot Embossing

**DOI:** 10.3390/mi8010029

**Published:** 2017-01-23

**Authors:** Lucas Abia Hof, Xin Guo, Minseok Seo, Rolf Wüthrich, Jesse Greener

**Affiliations:** 1 Department of Mechanical and Industrial Engineering, Concordia University, Montreal, QC H4B 1R6, Canada; l_hof@encs.concordia.ca; 2 FlowJEM Inc., 80 St. George Street, Toronto, ON M5S 3H6, Canada; sguo@flowjem.com (X.G.); info@flowjem.com (M.S.); 3 Posalux SA, 18, Fritz Oppliger, CH-2504 Biel/Bienne, Switzerland; rolf.wuthrich@concordia.ca or contact@posalux.com; 4 Département de Chimie, Université Laval, Québec, QC G1V 0A6, Canada

**Keywords:** micro-fabrication, hot embossing, micro-machining, microfluidics, glass, thermoplastics, MEMS, spark assisted chemical engraving

## Abstract

As the field of microelectromechanical systems (MEMS) matures, new demands are being placed on the microfabrication of complex architectures in robust materials, such as hard plastics. Iterative design optimization in a timely manner—rapid prototyping—places challenges on template fabrication, for methods such as injection moulding and hot embossing. In this paper, we demonstrate the possibility of using spark assisted chemical engraving (SACE) to produce micro patterned glass templates. The direct, write-based approach enabled the facile fabrication of smooth microfeatures with variations in all three-dimensions, which could be replicated by hot embossing different thermoplastics. As a proof of principle, we demonstrated the technique for a high glass transition temperature polycarbonate. Good fidelity over more than 10 cycles provides evidence that the approach is viable for rapid prototyping and has the potential to satisfy commercial-grade production at medium-level output volumes. Glass imprint templates showed no degradation after use, but care must be taken due to brittleness. The technique has the potential to advance microfabrication needs in academia and could be used by MEMS product developers.

## 1. Introduction

Micro electromechanical and micro optical electromechanical systems (MEMS and MOEMS) are poised to become a mainstream technology. One of the main developmental hurdles to overcome is devising new methods for fast and error-free micropatterning in plastics [1], which are among the fastest growing materials for industrial and clinical MEMS components [2]. Generally, fabrication techniques are either (i) sequential, such as milling, printing and ablation or (ii) template-based, such as casting, injection moulding and hot embossing, which create all features at the same time. Sequential methods can provide certain advantages, such as the straight-forward formation of three-dimensional features and a seamless design to prototyping workflow. However, they are not suitable for either high-volume requirements or even low-volume outputs, if the designs are too complex or high-density. On the other hand, moulding approaches that use a template, can be fast and accurate and are, therefore, more likely to be suitable for market-relevant volume demands. In hot embossing, a polymer is heated to its glass transition temperature (*T*_g_) and then formed into shapes based on contact with a microstructured template surface. It is similar to injection moulding, but differs in that complete melting is not required, which reduces energy consumption and residual thermal stress [3,4,5,6,7]. The replication process is straight forward, whereas the challenge lies in the fabrication of the imprint template. Typically, commercially available imprint templates are fabricated in metals such as nickel, using one or a combination of the following techniques: mechanical machining [8], laser ablation [9] and/or electroforming [8,9,10]. Such templates are advantageous in terms of their durability and thermal properties, but they are expensive and time-consuming to make. To date, the growth of hard plastics in final MEMS devices has only been possible due to the economies of scale that are realized in high-volume outputs, which can spread the cost of template development over many units. However, prototyping by hot embossing is still prohibitively expensive for complex designs requiring many iterations. Prototyping with lower cost techniques, such as casting polydimethylsiloxane (PDMS), is an option, but this will introduce new problems when switching fabrication modalities. Approaches for reducing the fabrication time and cost of the imprint templates by photolithography or by electroplating nickel through adhesive masks have been demonstrated, but both techniques have some limitations when considering the design aspect ratio and operating temperatures or feature resolution, respectively [4,11]. Furthermore, most of these approaches impose restrictions on the complexity of the design in the third dimension. So-called 2.5D templates can be achieved in multi-step fabrication processes, but this is time consuming, and requires specialized procedures. Recently, a new approach for producing 3D microfeatures in microfluidic channels has been shown, based on the control of embossing parameters to achieve controlled “partial” embossing, but the technique is still new and requires optimization [12]. The fabrication of imprint templates in new materials can open the door for rapid prototyping by hot embossing and a seamless transition to production-level runs for developed designs.

Literature reports on glass as an imprint template for polymer embossing are quite limited [13,14]. One of the main reasons that glass has not been considered a viable candidate for microembossing is due to the difficultly in its microscale patterning. Thermal etching based on femto-second laser processing can create features with a sub-micron resolution, but the equipment required is expensive and involves extensive set-up optimization. The preferred method for micropatterning glass is wet or dry chemical etching techniques. However, etching rates are slow and realizing 3D structures is not straightforward [15]. Mechanical machining is complicated due to the hardness and brittleness of glass. It is also difficult to achieve high-aspect ratio structures and is slow, costly and results in poor surface quality [16]. Alternative machining approaches, such as glass moulding techniques (e.g., glass reflow), make use of a high temperature step to reflow the glass into a micro-patterned silicon mould. However, multiple fabrication steps are needed (heating, lithography, patterning, vacuum cavities), processing times are very long and narrow patterns are complicated to achieve [17,18,19].

Recently, spark assisted chemical engraving (SACE) has been demonstrated as a promising approach for rapidly generating smooth, high aspect ratio 3D micro-structures in glass [16]. In this approach, a voltage is applied between a tool microelectrode and counter electrode, which are immersed in an electrolyte solution [16,20]. The electrolysis of water under an electrical potential (DC or pulsed voltage), causes bubble formation around the tool electrode. An electrical discharge passes through the gas, and is largely localized to its tip, where the electric field is highest. Thus, the machining of nearby glass surfaces becomes possible due to thermally promoted hydroxide ions, which cause the localized breaking of Si–O–Si bonds [16,21]:

2NaOH + SiO_2_ → Na_2_SiO_3_ + H_2_O (1)

More detailed information on the etching of silica glass in the presence of OH-containing electrolytes, can be found in the literature [22,23,24].

With the proper choice of processing parameters (temperature [21], voltage [25], electrolyte type, and its concentration [16,20]), SACE can be optimized to rapidly produce smooth micro-structured glass features with shape control in three-dimensions. Relative to other techniques, it has high machining speeds (cm·s^−1^), a range of target materials (glasses [26], ceramics [27], and composite materials [28]) and is low-cost [16]. Thus, for the purpose of this work, SACE was selected as a method for producing imprint templates in glass for the testing and development of a new method for microfabrication applications requiring rapid prototyping in thermoplastics by hot embossing. The utility of glass templates was tested through the replication of micro features, including drill holes and channels with different depths and surface finishes.

## 2. Materials and Methods

Consumables included polycarbonate (PC) (Lexan 9034-112, Sabic Polymershapes, Brampton, ON, Canada), polypropylene (PP) (PolyPrime, Coquitlam, BC, Canada), cycloolifin polymer (COP) (Zeon, Zeonex, Louisville, KY, USA), polymethyl methacrylate (PMMA) (Plaskolite, Optix, Columbus, OH, USA), polystyrene (PS) (McMaster Carr, Elmhurst, IL, USA), polydimethyl siloxane (PDMS) (Sylgard184, Dow Corning, Midland, MI, USA), glass (borosilicate, square 50.8 mm, thickness = 3.175 mm, Corning Inc., Corning, NY, USA), potassium hydroxide (KOH Cas 1310-58-3, Cayman Chemical Company, Ann Arbor, MI, USA), tool-electrodes (tungsten carbide cylinders, diameter = 100 µm, Posalux SA, Biel/Bienne, Switzerland), and Trichloro (1*H*,1*H*,2*H*,2*H*-perfluorooctyl) silane (Sigma-Aldrich, Oakville, ON, Canada).

Milling designs were created using computer-aided design software (AutoCAD 2015, AutoDesk San Rafael, CA, USA). Glass machining was conducted using an industrial SACE system (Microfor SACE, Posalux SA, Biel/Bienne, Switzerland) and a home-build lab SACE set-up (SACE1, Electrochemical Green Engineering Group, Concordia University, Montreal, QC, Canada). A universal motion controller (XPS-C4, Newport, Irvine, CA, USA) was used to program the *x*,*y*,*z* axes. *z*-Direction control was achieved by a proportional–integral–derivative (PID) control loop using an optical sensor and voicecoil actuator on the machining head.

Embossing was accomplished using a converted hydraulic press (Model 3851-C Carver Inc., Wabash, IN, USA) with ±1 °C temperature control, compressed gas cooling was applied to the top and bottom platens, and ±0.05 MPa pressure control was applied between the platens. Embossing was conducted inside a custom compression chamber that was placed between the top and bottom platens, with leads for gas exchange and vacuum control. The temperatures inside the embossing chamber were determined to be 4–6 °C lower than the platen set values. No determination of the real pressures applied to the embossing tool and plastic substrate was made, but it was estimated to be between 0.2 and 0.4 MPa lower than the value determined by the external pressure gauge.

Evaluation of the microfeatures on the embossing template and embossed polymer substrate was conducted using optical profilometry (Veeco NT 1100, Veeco, Plainview, NY, USA) and optical microscopy (Olympus BX41, Olympus, Center Valley, PA, USA) was combined with the application of a charge-coupled device (CCD) camera (EvolutionTM VF, Media Cybernetics Inc., Silver Spring, MD, USA) using ImagePro software. Root mean square (RMS) roughness was conducted on optical profile images using the plugin “Analyse Stripes” in the open source software ImageJ (software developed by contributors worldwide).

## 3. Results and Discussion

### 3.1. Mould Fabrication Using SACE

#### 3.1.1. System Operation

**Figure 1:**
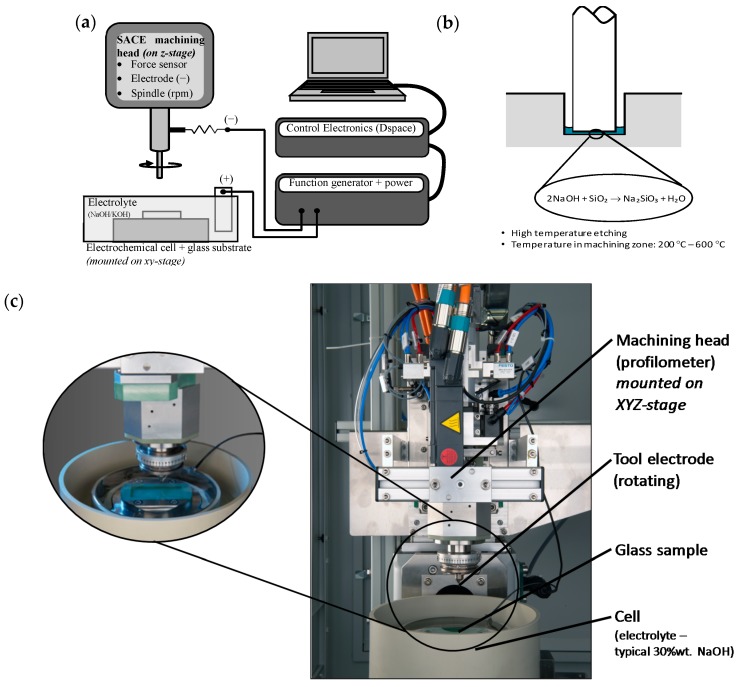
(**a**) Laboratory spark assisted chemical engraving (SACE) versatile glass micromachining setup. (**b**) Chemical mechanism responsible for the localized degradation of the SiO_2_ network at the SACE tool tip (blue). (**c**) Image of the industrial Microfor SACE machine (Posalux SA) used in this work with inset showing a close up of the machining zone.

The electrochemical cell, containing the electrolyte (typical NaOH or KOH), counter electrode (stainless steel 316L, Advent Research Materials, Oxford, UK), and the target glass substrate, was mounted on an *x*,*y* stage (resolution, 1 µm), whereas the axial movement of the electrode tool (tungsten carbide) was performed on a high precision *z*-stage (resolution, 1 µm). High accuracy tool tips, with diameters of 100 µm, were used to enhance machining accuracy and resolution. In addition, tool rotation was implemented by a dc brushless motor, added to the *z*-axis stage to improve the uniformity of electrolyte flow around the tool, thus increasing the quality of the machined features. When high voltages (around 30 V) were applied between the counter electrode and the tool, the bubbles evolving around the tool electrode coalesced into a gas film, and electrical discharge was emitted from the tool and passed through the film to the electrolyte, as illustrated in Figure 1. Usually, the voltage is pulsed between a high voltage (between 28 and 40 V) and a lower voltage (typically 17.5 V), with a period = 2.6 ms, and a duty cycle = 96.15%, in order to limit temperature buildup. The machining conditions for the SACE process of glass mould fabrication are denoted in Table 1.

#### 3.1.2. Control over 3D Structure

**Figure 2:**
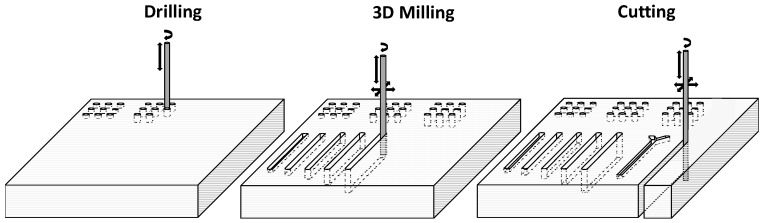
A graphic highlighting 3D drilling, milling, and cutting machining operations performed on the same glass substrate.

The developed industrial SACE machine offered high precision glass micro-drilling, micro-milling, micro-cutting and micro 3D machining operations (Figure 2), while leaving the glass surface intact to allow subsequent glass-to-polymer templating. The implementation of a force-sensitive machining head with force-feedback algorithms could detect and maintain forces as low as 1 mN. This enabled the usage of narrow machining tools without bending or breaking, and secured its usage as an accurate profilometer for measuring machined features within the same set-up to an accuracy of 1 µm, enabling continuous three dimensional control.

#### 3.1.3. Surface Smoothness for Hot Embossing Applications

The SACE milling technique can be optimized to improve the surface uniformity of the etched features. This enhanced surface quality was achieved on the industrial Microfor SACE machine (Figure 1c). It is proposed that a smoothed edge will have two positive effects for the present application of hot embossing. First, reduced surface area on the smoothed feature side walls, will reduce the friction between the template and plastic substrate during de-embossing, thereby lowering the force required to separate them. Second, there will be a reduction in the number of potential fracture sites on a smooth glass surface, in comparison to a rough one. Together, these factors should strongly improve the quality of the embossed features and the longevity of the imprint template, by reducing the chance of damage during de-embossing.

For the purpose of this study, SACE process parameters were chosen in order to reduce the surface roughness of the cut side walls. It should be noted that surface smoothness optimization experiments are ongoing, and the results presented in this paper are based on preliminary results produced when using the Microfor SACE machine (Posalux SA). To evaluate the smoothness of the machined side wall, a measurement was made for the surface distance index (SDI), which is defined as:

SDI = *d_s_*/*d* (2)

where *d_s_* is the total path length, including wall roughness, between two points along the machined edge and *d* is the distance of the straight line between the end points.

**Figure 3:**
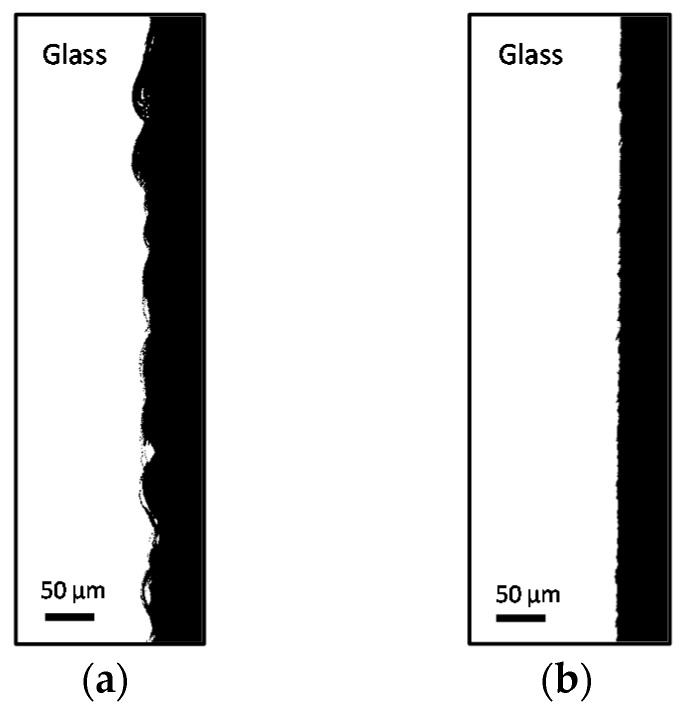
Top-view microscope images of example surfaces after SACE milling: a cut made with the lab set-up (**a**) and a cut made on the industrial Microfor SACE machine (Posalux SA) (**b**). Scale bars are in 50 µm.

SACE optimization reveals a SDI improvement of 0.089 (i.e., about 9%), based on a measured SDI of 1.098 for the fast rough cut (Figure 3a), and an SDI of 1.009 for the optimized cut (Figure 3b). These correspond to a RMS surface roughness of 3.77 and 1.13 µm, respectively.

#### 3.1.4. Fabrication and Evaluation of a Glass Imprint Template

**Figure 4:**
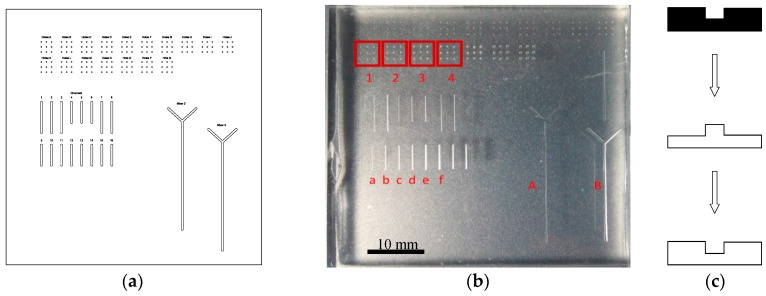
A schematic from the CAD file (**a**) that was used to produce the glass template by SACE milling for this work (**b**). Highlighted regions include drill arrays (red box 1–4) and trenches (“a”–“f”, “A”, “B”). A schematic showing a double replication procedure of the original template (black) for producing positive replicates in PDMS (white) (**c**).

To demonstrate the approach for hot embossing, glass imprint template moulds were fabricated, containing features for analysis, including drilled holes and channels (Figure 4). Additionally, other features were added to subsequent glass templates, including spiral patterns, rings, and crosses. Due to the flexibility in prototyping, the features had a range of depths, ranging from 50 to 750 µm on the same glass substrate, leading to aspect ratios of between 0.2 and 3.0. While the technique has been used to create aspect ratios of 10 or higher [16], we chose to work with smaller values in order to demonstrate the technique without complications related to de-embossing high aspect ratio features. For a comparison of the feature quality after a polishing step, a series of channels were fabricated using the same protocol as above.

Features were analysed using a combination of optical profilometry and cross-sections in replicates of the glass mould, which were easier to cut and examine. To achieve the latter, a double replication process was undertaken, resulting in a positive PDMS part which could be cross-sectioned with a blade and viewed with a regular transmission light microscope. The replication process started by casting PDMS against the glass mould. This formed a negative of the glass mould. Then, a second replica was formed by casting PDMS against the first, to create a positive replica. In each replication stage, the casting surface was silonized to enable separation of the parts.

#### 3.1.5. Time to Create a Stamp

The time required to create a channel feature on the glass substrate was not a key optimization parameter in this study. Instead, focus was placed on achieving features with low surface roughness (<several microns). Nevertheless, it is instructive to evaluate this parameter as a benchmark for future optimization. As an example, the features a, d, and f, shown in Figure 4b, required 21 min, 23 min, and 25 min, respectively, based on their length (*L* = 5 mm), number of passes (depth-of-cut per pass = 50 µm) required (*P*_a_ = 2, *P*_d_ = 8, *P*_f_ = 12) and additional optimization steps. Therefore, it is reasonable to conclude that a complete new master, with significantly higher complexity, could be fabricated in under a day. This is nearly an order of magnitude faster than current methods, before having considered the arbitrary SACE ability to make 3D features at the same time. Chemical methods, such as deep reactive ion etching (DRIE) etching and wet etching, have very low etching rates, with average rates of 0.01 and 0.1 µm·s^−1^, respectively. Moreover, they need cleanroom facilities and require additional sophisticated masking steps. In addition, wet etching (standard masking procedures) limits the achievable aspect ratio to one. Mechanical methods, such as drilling and milling (feed rates around 100 µm·s^−1^), and powder blasting (feed rates around 1 µm·s^−1^), have feed rates which are similar to, or faster than, SACE technology. However, machined surfaces suffer from high surface roughness and microcracks. It is uncertain whether such glass surfaces could work for repetitive cycling through hot embossing steps. Thermal processes, such as ultra-short laser technologies (e.g., Femto-second laser), have increased feed rates (around 10–10,000 µm·s^−1^), however, these technologies need extensive set-up optimization and are expensive. The use of a hybrid technology such as SACE seems to be an appropriate choice for the rapid prototyping of glass moulds.

### 3.2. Use of Glass Moulds as Imprint Templates for Microfabrication

#### 3.2.1. Glass Properties

Table 2 compares relevant glass properties with other materials used for hot embossing templates. The most widely used material is Ni, which has a low thermal expansion, high thermal conductivity, and good mechanical properties. However, the cost and time required to produce Ni imprint templates is substantial, rendering it ineffective for prototyping. In searching for alternative materials for embossing templates, researchers have looked to polymeric materials, such as PDMS, photoresists and epoxy resins. The major drawback of these materials includes poor thermal conductivity and high linear expansion coefficients. Their hardness is also generally much lower than that of Ni, even with process optimizations [29]. Low hardness can lead to blemishes in the template from microscopic dust and debris, thereby limiting their practical life-times. In addition, fabricating 3D structures is challenging.

Glass is an interesting option, due to its good thermomechanical properties. First, glass is superior to all other options in terms of its low thermal expansion. Hardness prevents degradation of the template [30]. Glass and polymer-based template materials all suffer from low thermal conductivity. This means that overall embossing times can be longer than those of metal stamps. However, the difference is in terms of minutes, which is not significant for low and medium production levels. Fracture toughness is related to material brittleness and is a measure of a material’s ability to resist cracking. It is one of the most relevant mechanical properties for hot embossing. In glass, this is affected by ion type and concentration [31,32]. The low fracture toughness of glass is its greatest drawback for hot embossing applications. However, as discussed later, this problem can be overcome by following certain fabrication protocol.

#### 3.2.2. Embossing Protocol

**Figure 5:**
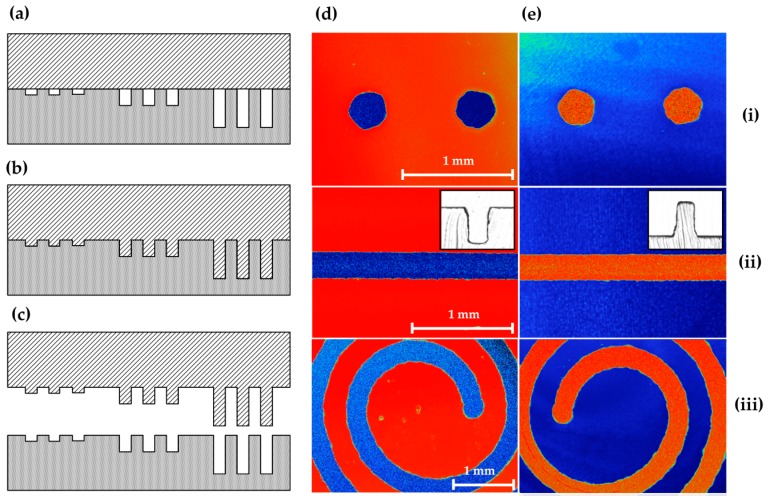
The process of embossing includes (**a**) putting a polymer sheet in light contact with the glass template while the system temperature is elevated to *T*_e_. (**b**) After stabilization of temperature, embossing pressure is applied and the heated polymer conforms to the template bas-relief features. (**c**) After cooling to *T*_d_, the master is separated from the patterned polymer. Optical profilometry of (**d**) features on the glass template and (**e**) the embossed PC substrate for vertical drill holes (**i**), straight trenches (**ii**), and spiral pattern (**iii**). Red and blue colours show raised and recessed surfaces, respectively. Inset figures for straight trenches in glass and the corresponding embossed PC were acquired from microscopy of cross-sections. Scale bars for template images (**d**) are each 1 mm and common for the corresponding image in (**e**).

Due to the non-zero water absorption by most thermoplastics used here, films (0.5 mm) were first dehydrated before embossing, by pre-heating in an oven at roughly 80 °C for *t*_d_ = 40–80 min. The glass imprint template was then loaded with its features facing up, onto the bottom side of the custom embossing chamber, which was itself on the bottom platen of the hot press. The thermoplastic material was placed on top of the glass template (Figure 5a). Following this, the chamber was closed and a vacuum was applied, while the system heated to an embossing temperature *T*_e_. An embossing pressure (*p*) was then applied, which forced the heated polymer into the machined cavities in the glass template (Figure 5b). After two minutes, the system temperature was reduced to the de-embossing temperature (*T*_d_), followed by the breaking of the vacuum and the separation of the moulded plastic from the template (Figure 5c). Relevant thermal properties, as well as the embossing parameters for different thermoplastic materials, are given in Table 3.

To demonstrate the robustness of the technique, we carried out replication on PC, which had the highest *T*_g_ of the test materials used in this study. Figure 5d,e show the images of certain features on the glass template and the results from the embossed PC. The glass template features included (i) drill holes, (ii) channels and (iii) curved shapes (Figure 5d). All template features (i) and (iii) were fabricated with a home-build instrument, whereas (ii) was produced on the commercial SACE instrument to achieve low surface roughness.

### 3.3. Repetitive Embossing Using a SACE Imprint Template

Lastly, we determined the fidelity of the technique under repeated use. Generally, the number of fabrication cycles during prototyping is three [46]. In order to demonstrate that glass moulds can exceed the durability required for prototyping purposes, 11 embossing cycles were undertaken on PC, for three designs (from Figure 4), each with different dimensions. After each embossing cycle, the feature height (*h*), width (*w*) and root mean surface roughness (*Rq*), were measured, from optical profilometry results. Table 4 shows the summarized results for the embossed substrates after cycles one, five and eleven. These are compared to the same parameters in the glass imprint template. Reproducibility was good for smaller features, but became less certain for larger structures. While optimization is required, we note that lower tolerances may be acceptable for larger features.

### 3.4. Discussion

The role of glass in MEMS and MOEMS has typically been limited to its integration into the final device-level product [15]. Until now, the idea of using glass as a template surface for high energy fabrication processes like hot embossing, has been presented as little more than a curiosity. Of the two major challenges in accomplishing this, brittleness and difficultly in surface patterning, it is our opinion that it is the latter which was the true road block. With new glass microfabrication methods like SACE, this can be overcome. The question then turns to proper utilization of the glass template, to ensure suitable longevity for MEMS prototyping output levels, at least. Here, we discuss some guidelines that were developed for achieving this goal, followed by suggested avenues for further development.

First, the concern of brittle glass templates that can crack during use, is both justified and avoidable. Contrary to our initial direction, however, thick glass templates can actually cause more problems than they solve. Apart from the practical problems of incorporating a thicker-than-usual template into embossing apparatus, the low thermal conductivity of thick glass samples necessitates longer warm-up times. Failure to do so can result in improper embossing, due to lower-than-expected temperatures. Worse, the application of pressure before the temperature has been stabilized can result in template failure due to temperature gradients. Furthermore, the common application of a flexible pad, used to redistribute pressure gradients from misaligned platens, should be avoided with glass templates. In fact, pressure gradients are acceptable as long as the template is held fast and supported at all points. Thus, placing the template against a solid, smooth surface, such as the embossing chamber floor, is ideal. In this case, dust and other small particles must be carefully removed.

In order to complete a rapid transition to the volume-level production after prototyping , the two phases should mirror each other as much as possible. Using hot embossing during prototyping is an asset in this case, but the imprint templates should also be similar. For example, taking advantage of the 3D writing capabilities of SACE for prototypes, will not translate well to volume production phases, if a more robust template material is required, necessitating its redevelopment of the fabrication protocol with new methods. Here, we look forward to new opportunities for using a SACE-produced glass template as a master from which to directly produce more robust templates, such as the casting of epoxies. Lastly, the technique demonstrated here is well-suited for the accurate removal of small amounts of glass material. As the replication process produces inverse geometries, protrusions are easily fabricated by embossing, whereas depressions are more difficult. Identifying and overcoming these and other limitations should be contemplated in order for glass imprint templates to find their place amongst the new wave of emerging microfabrication techniques.

## 4. Conclusions

In this work, the use of SACE milled glass templates for the embossing of microstructures in thermoplastics was demonstrated. This technique had the advantage of rapidly and accurately introducing features into glass substrates, with good control over all three dimensions. Optimization of the technique for generating smooth surfaces was demonstrated using a new commercially available SACE machine. With proper care during embossing, the use of glass templates provides a viable alternative to expensive and time-consuming template fabrication methods. Here, it is demonstrated that repeated embossing cycles (*n* > 10) generate samples with minimal variation in their features from run-to-run, especially for small features. This preliminary work opens the door to further developments that can significantly reduce the time and cost required for the production of microfabricated plastic parts.

## Figures and Tables

**Table 1:** SACE machining conditions.

Feature	Machining Mode	Electrolyte	Pulsed Voltage Input			
			High Level	Low Level	Period	Duty Cycle
Channels/lines	Constant depth-of-cut (50 µm) × *n*	20 wt % KOH	36 V	17.5 V	2.6 ms	96.15%
Holes	Gravity feed drilling	20 wt % KOH	36 V	17.5 V	2.6 ms	96.15%

**Table 2:** Comparison of relevant properties for different hot embossing template materials.

Material	Linear Temp. Expansion Coefficient (µm∙(m∙k)^−1^)	Thermal Conductivity (W∙(m∙k)^−1^)	Hardness (GPa)	Fracture Toughness (MPa∙m^1/2^)	Tensile Strength (MPa)	Compressive Strength (MPa)
Glass borosilicate (toughened) [33]	4.0	1.05	6.2 [34,35]	0.7 [36]/2 [37]	30/200	1000
PDMS (Stylgard 184) [38]	310	0.15 [39]	N/A ^1^	-	7 [40]	2–50
Photoresist (SU-8 series) [41]	52	0.2	0.3 [42]	-	73	-
Epoxy resins [43]	-	-	N/A ^2^ [43,44]	400	70 [45]	-
Ni	13.0	91	6.3–11.8	100–150	-	-

^1^ The elastomer PDMS has a measured hardness of 50 in the Shore A scale, a scale ranging from 1 to 100 for the softest polymer materials; ^2^ the resin elastomer Conapoxy has a measured hardness of 90 in the Shore D scale, a scale ranging from 1 to 100 for the hardest polymer based materials.

**Table 3:** Material properties and embossing conditions for materials used in this study.

Material	Material Properties		Embossing Conditions			
	*T*_g_^1^ (°C)	*T*_m_^1^ (°C)	*t*_d_^2^ (min)	*T*_e_/*T*_d_^3^ (°C)	*p* ^4^ (PSI)	*t*_e_^5^ (min)
COP	138	210	50–100	150/130	70	5
PMMA	113	160	75–90	145/80	130	5
PC	149	155	40–80	175/145	150	5
PS	101	240	70–80	115–125/100	120–130	5
PP	−6	164	N/A	145/100	150	5

^1^ *T*_g_ and *T*_m_ are the glass transition temperature and melting point, respectively; ^2^ *t*_d_ is the material dehydration time as recommended by the manufacturer; ^3^ *T*_e_ and *T*_d_ are the embossing and de-embossing temperatures, respectively; ^4^ *p* is the embossing pressure which is held for 2 min; ^5^ *t*_e_ is the temperature stabilization time at the embossing temperature before pressure is applied.

**Table 4:** Embossing results for substrates after embossing process cycles one, five and eleven.

Feature		Glass Template				1st Embossing			5th Embossing			11th Embossing			Feature Statistics Line a, d, f
Name	*d*	Image	*d*	*w*	*Rq*	*d*	*w*	*Rq*	*d*	*w*	*Rq*	*d*	*w*	*Rq*	Average *d*, *w*, *Rq*
Line a	50	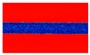	42	251	6.9	42	237	5.3	42	239	3.5	41	239	3.1	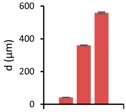
Line d	350	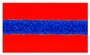	361	318	8.8	362	316	8.8	358	313	5.0	359	312	8.4	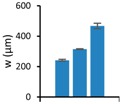
Line f	550	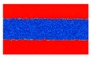	565	457	11.6	556	471	18.5	560	491	9.4	554	451	6.2	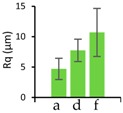

All dimensions (*d, w, Rq*) are in micrometers. Image resolution in the *z*-direction was ±4 µm.

## References

[1] Berthier E., Young E.W.K., Beebe D. (2012). Engineers are from PDMS-land, biologists are from polystyrenia. Lab Chip.

[2] Yole Development Market and Technology Trends for Microfluidic Applications—21 September 2011.

[3] Cameron N.S., Roberge H., Veres T., Jakeway S.C., John Crabtree H. (2006). High fidelity, high yield production of microfluidic devices by hot embossing lithography: Rheology and stiction. Lab Chip.

[4] Greener J., Li W., Ren J., Voicu D., Pakharenko V., Tang T., Kumacheva E. (2010). ESI: Rapid, cost-efficient fabrication of microfluidic reactors in thermoplastic polymers by combining photolithography and hot embossing. Lab Chip.

[5] Leech P.W. (2009). Hot Embossing of Microchannels in Cyclic Olefin Copolymer. Camb. J. Online.

[6] Gates B.D., Xu Q., Love J.C., Wolfe D.B., Whitesides G.M. (2004). Unconventional nanofabrication. Annu. Rev. Mater. Res..

[7] Yao D., Nagarajan P., Li L., Yi A.Y. (2007). A two-station embossing process for rapid fabrication of surface microstructures on thermoplastic polymers. Polym. Eng. Sci..

[8] Schaller T., Bohn L., Mayer J., Schubert K. (1999). Microstructure grooves with a width of less than 50 μm cut with ground hard metal micro end mills. Precis. Eng..

[9] Shiu P.P., Knopf G.K., Ostojic M., Nikumb S. (2008). Rapid fabrication of tooling for microfluidic devices via laser micromachining and hot embossing. J. Micromech. Microeng..

[10] Shibata T., Takahashi Y., Kawashima T., Kubota T., Mita M., Mineta T., Makino E. (2008). Micromachining of electroformed nickel mold using thick photoresist microstructure for imprint technology. Microsyst. Technol..

[11] Novak R., Ranu N., Mathies R.A. (2013). Rapid fabrication of nickel molds for prototyping embossed plastic microfluidic devices. Lab Chip.

[12] Debono M., Voicu D., Pousti M., Safdar M., Young R., Kumacheva E., Greener J. (2016). One-step fabrication of microchannels with integrated three dimensional features by hot intrusion embossing. Sensors.

[13] Zhang L., Gu F., Tong L., Yin X. (2008). Simple and cost-effective fabrication of two-dimensional plastic nanochannels from silica nanowire templates. Microfluid. Nanofluid..

[14] Niino H., Ding X., Kurosaki R., Narazaki A., Sato T., Kawaguchi Y. (2004). Imprinting by hot embossing in polymer substrates using a template of silica glass surface-structured by the ablation of LIBWE method. Appl. Phys. A.

[15] Iliescu C., Taylor H., Avram M., Miao J., Franssila S. (2012). A practical guide for the fabrication of microfluidic devices using glass and silicon. Biomicrofluidics.

[16] Wüthrich R., Ziki J.D.A., Wüthrich R., Ziki J.D.A. (2015). Micromachining Using Electrochemical Discharge Phenomenon.

[17] Van Toan N., Toda M., Ono T. (2016). An investigation of processes for glass micromachining. Micromachines.

[18] Liu J.W., Huang Q.A, Shang J.T., Tang J.Y. Micromachining of Pyrex7740 Glass for Micro-Fluidic Devices. Proceedings of the 14th International Conference on Miniaturized Systems for Chemistry and Life Sciences.

[19] Haque R.U.M., Wise K.D. (2013). A glass-in-silicon reflow process for three-dimensional microsystems. J. Microelectromech. Syst..

[20] Wüthrich R., Fascio V. (2005). Machining of non-conducting materials using electrochemical discharge phenomenon—An overview. Int. J. Mach. Tools Manuf..

[21] Abou Ziki J.D., Hof L.A., Wüthrich R. (2015). The machining temperature during spark assisted chemical engraving of glass. Manuf. Lett..

[22] Le Bourhis E. (2014). Glass, Mechanics and Technology.

[23] Zumdahl S.S., DeCoste D.J., White A. (2010). Introductory Chemistry.

[24] Boyd D.C., Danielson P.S., Thompson D.A., Velez M., Reis S.T., Brow R. (2004). Glass. Kirk-Othmer Encyclopedia of Chemical Technology.

[25] Zheng Z.-P., Su H.-C., Huang F.-Y., Yan B.-H. (2007). The tool geometrical shape and pulse-off time of pulse voltage effects in a Pyrex glass electrochemical discharge microdrilling process. J. Micromech. Microeng..

[26] Cao X.D., Kim B.H., Chu C.N. (2009). Micro-structuring of glass with features less than 100 μm by electrochemical discharge machining. Precis. Eng..

[27] Chak S.K., Venkateswara Rao P. (2008). The drilling of Al_2_O_3_ using a pulsed DC supply with a rotary abrasive electrode by the electrochemical discharge process. Int. J. Adv. Manuf. Technol..

[28] Liu J.W., Yue T.M., Guo Z.N. (2010). An analysis of the discharge mechanism in electrochemical discharge machining of particulate reinforced metal matrix composites. Int. J. Mach. Tools Manuf..

[29] Kim M., Moon B.-U., Hidrovo C.H. (2013). Enhancement of the thermo-mechanical properties of PDMS molds for the hot embossing of PMMA microfluidic devices. J. Micromech. Microeng..

[30] Jaszewski R.W., Schift H., Gobrecht J., Smith P. (1998). Hot embossing in polymers as a direct way to pattern resist. Microelectron. Eng..

[31] Soga N. (1985). Elastic moduli and fracture toughness of glass. J. Non Cryst. Solids.

[32] Yamane M., Mackenzie J.D. (1974). Vicker’s Hardness of glass. J. Non Cryst. Solids.

[33] Saint Gobain Glass Glass Physical Properties. http://uk.saint-gobain-glass.com/trade-customers/physical-properties.

[34] Wilantewicz T.E., Varner J.R. (2008). Vickers indentation behavior of several commercial glasses at high temperatures. J. Mater. Sci..

[35] Denry I.L., Holloway J.A. (2004). Elastic constants, Vickers hardness, and fracture toughness of fluorrichterite-based glass-ceramics. Dent. Mater..

[36] Matzke H., Toscano E., Routbort J., Reimann K. (1986). Temperature dependence and fracture toughness and elastic moduli of a waste glass. J. Am. Ceram. Soc..

[37] Petit F., Sartieaux A.C., Gonon M., Cambier F. (2007). Fracture toughness and residual stress measurements in tempered glass by Hertzian indentation. Acta Mater..

[38] Johnston I.D., McCluskey D.K., Tan C.K.L., Tracey M.C. (2014). Mechanical characterization of bulk Sylgard 184 for microfluidics and microengineering. J. Micromech. Microeng..

[39] MIT Material Properties PDMS. http://www.mit.edu/~6.777/matprops/pdms.htm.

[40] Greer A.I.M., Vasiev I., Della-Rosa B., Gadegaard N. (2015). Fluorinated ethylene–propylene: A complementary alternative to PDMS for nanoimprint stamps. Nanotechnology.

[41] Microchem Material Properties. http://www.microchem.com/pdf/SU-83000DataSheet.pdf.

[42] Hammacher J., Fuelle A., Flaemig J., Saupe J., Loechel B., Grimm J. (2008). Stress engineering and mechanical properties of SU-8-layers for mechanical applications. Microsyst. Technol..

[43] Jena R.K., Yue C.Y., Yun K.X. (2014). Effect of a CNT based composite micromold on the replication fidelity during the microfabrication of polymeric microfluidic devices. RSC Adv..

[44] Cytec Idustries Inc. Conapoxy FR-1080. http://www.needfill.co.kr/cd/FR-1080.html.

[45] Ellsworthadhesives Epoxy Resins Material Properties. http://www.ellsworthadhesives.co.uk/media/wysiwyg/files/cytec/CytecElectronicsBrochure-EU.pdf.

[46] Statistics based on 2011–2016 order statistics from FlowJEM Inc..

